# Sleep duration and risk of overall and 22 site‐specific cancers: A Mendelian randomization study

**DOI:** 10.1002/ijc.33286

**Published:** 2020-09-14

**Authors:** Olga E. Titova, Karl Michaëlsson, Mathew Vithayathil, Amy M. Mason, Siddhartha Kar, Stephen Burgess, Susanna C. Larsson

**Affiliations:** ^1^ Unit of Medical Epidemiology, Department of Surgical Sciences Uppsala University Uppsala Sweden; ^2^ MRC Cancer Unit University of Cambridge Cambridge UK; ^3^ British Heart Foundation Cardiovascular Epidemiology Unit, Department of Public Health and Primary Care University of Cambridge Cambridge UK; ^4^ National Institute for Health Research Cambridge Biomedical Research Centre University of Cambridge and Cambridge University Hospitals Cambridge UK; ^5^ MRC Integrative Epidemiology Unit, Bristol Medical School University of Bristol Bristol UK; ^6^ Department of Public Health and Primary Care University of Cambridge Cambridge UK; ^7^ MRC Biostatistics Unit University of Cambridge Cambridge UK; ^8^ Unit of Cardiovascular and Nutritional Epidemiology Institute of Environmental Medicine, Karolinska Institutet Stockholm Sweden

**Keywords:** cancer, Mendelian randomization, single‐nucleotide polymorphisms, sleep

## Abstract

Studies of sleep duration in relation to the risk of site‐specific cancers other than breast cancer are scarce. Furthermore, the available results are inconclusive and the causality remains unclear. We aimed to investigate the potential causal associations of sleep duration with overall and site‐specific cancers using the Mendelian randomization (MR) design. Single‐nucleotide polymorphisms associated with the sleep traits identified from a genome‐wide association study were used as instrumental variables to estimate the association with overall cancer and 22 site‐specific cancers among 367 586 UK Biobank participants. A replication analysis was performed using data from the FinnGen consortium (up to 121 579 individuals). There was suggestive evidence that genetic liability to short‐sleep duration was associated with higher odds of cancers of the stomach (odds ratio [OR], 2.22; 95% confidence interval [CI], 1.15‐4.30; *P* = .018), pancreas (OR, 2.18; 95% CI, 1.32‐3.62; *P* = .002) and colorectum (OR, 1.48; 95% CI, 1.12‐1.95; *P* = .006), but with lower odds of multiple myeloma (OR, 0.47; 95% CI, 0.22‐0.99; *P* = .047). Suggestive evidence of association of genetic liability to long‐sleep duration with lower odds of pancreatic cancer (OR, 0.44; 95% CI, 0.25‐0.79; *P* = .005) and kidney cancer (OR, 0.44; 95% CI, 0.21‐0.90; *P* = .025) was observed. However, none of these associations passed the multiple comparison threshold and two‐sample MR analysis using FinnGen data did not confirm these findings. In conclusion, this MR study does not provide strong evidence to support causal associations of sleep duration with risk of overall and site‐specific cancers. Further MR studies are required.

## Abstract 2

**What's new?**

With the exception of breast cancer, the impact of sleep duration on site‐specific cancer risk remains largely unknown. Here, using Mendelian randomization analysis, the authors investigated the causal role of sleep duration for overall cancer and for 22 site‐specific cancers in more than 367 000 UK Biobank participants. Suggestive associations were detected between certain cancer sites and genetic liability to short or long sleep duration or genetically predicted sleep duration. However, no statistically significant associations were identified between sleep duration and overall or site‐specific cancer risk, indicating that sleep duration does not have a causal influence on cancer.

**Abbreviations**
BMI: body mass index
CI: confidence interval
FDR: false discovery rate
GWAS: genome‐wide association study
IGF: insulin‐like growth factor
IVW: inverse variance‐weighted
MR: Mendelian randomization
OR: odds ratio
SNP: single‐nucleotide polymorphism

## 1 INTRODUCTION

Sleep is essential for maintaining optimal physiological processes. Sleep‐related problems are becoming increasingly prevalent in modern society, affecting 20% to 40% of the general population.^1^ During the last decade, increased attention has been paid to understanding to what extent impaired sleep patterns, including those characterized by habitual short‐sleep duration, are associated with various adverse health outcomes, such as obesity, type 2 diabetes, cognitive impairment, cardiovascular disease and all‐cause mortality.^2^ Growing evidence suggests that short‐ and long‐sleep duration may also be implicated in the development of cancer.^1^, ^3^, ^4^

Several observational studies have examined the association between short‐sleep (commonly defined as <7 hours/night) and long (commonly defined as ≥9 hours/night) sleep duration and cancer risk.^3^ An increased risk of colorectal, breast and lung cancers among short and long sleepers^5^, ^6^, ^7^ and an increased risk of stomach cancer^8^ and overall cancer among short sleepers^9^ have been reported by these observations. For example, a study of 23 620 middle‐aged participants adults found that individuals who on average slept less than 6 hours had 43% higher risk of cancer (846 cases during a mean follow‐up of 7.8 years) compared with those who slept 7 to <8 hours.^9^ The effect of sleep deprivation on tumor development has also been investigated in experimental studies in mice, and it has been shown that chronic sleep restriction impairs antitumor immune responses and increases growth rate in pulmonary metastasis.^10^ However, the results remain inconsistent as several epidemiological studies and meta‐analyses have found no association between sleep duration and risk of cancer.^3^, ^11^, ^12^ Based on available evidence, no clear consensus about the effect of sleep duration on cancer risk can be drawn as observational studies are susceptible to confounding and reverse causality.

Mendelian randomization (MR) is an epidemiologic technique that utilizes genetic variants that are reliably associated with potentially modifiable risk factors to determine their causal role for disease risk.^13^, ^14^ MR studies are less vulnerable to confounding, reverse causation bias and measurement error compared with conventional observational studies. We used the MR design to investigate the associations of short‐ and long‐sleep duration with overall cancer and 22 site‐specific cancers.

## 2 MATERIALS AND METHODS

### 2.1 Study population

This MR study is based on data from the UK Biobank study.^15^ This cohort enrolled around 500 000 adults, aged 37 to 73 years, from 22 assessment centers across the United Kingdom during 2006 and 2010. For this analysis, we limited the study population to individuals of European descent to reduce population stratification bias. After exclusion of related individuals (third‐degree relatives or closer), low genotype call rate (three or more standard deviations from the mean) and excess heterozygosity, 367 586 participants remained for analysis. The cancer outcomes were defined based on diagnosis codes, histology, self‐reported data (validated by interview with a trained nurse) and follow‐up information until March 2017 was used. Logistic regression with adjustment for age, sex, genotyping array and 50 genetic principal components was used to obtain the genetic‐cancer association estimates. The analyses were conducted under UK Biobank application 29 202. A replication analysis was performed using data from the FinnGen consortium (R3 data release; up to 121 579 individuals). Detailed description of the methods used by FinnGen can be found in its webpage (https://www.finngen.fi/fi).

### 2.2 Instrumental variable selection

We selected all single‐nucleotide polymorphisms (SNPs) previously shown to be associated with the sleep duration traits at the level of genome‐wide significance (*P* < 5 × 10^−8^) among 446 118 UK Biobank participants of European ancestry.^16^ Genetic association analysis in this study was performed in subjects of European ancestry using BOLT‐LMM (linear mixed models) and an additive genetic model adjusted for age, sex, 10 principal components of ancestry, genotyping array and genetic correlation matrix.^16^ Linkage disequilibrium (defined as *r* ^2^ > 0.1 in European populations) between SNPs was evaluated using LDlink.^17^ Given that the association between sleep duration and cancer risk may be nonlinear, we used SNPs associated with continuous sleep duration as well as with short and long sleep. The number of SNPs used were 27 for short‐sleep duration (n = 106 192 cases with <7 hours of sleep relative to 305 742 controls with 7‐8 hours of sleep), eight for long sleep (n = 34 184 cases with ≥9 hours of sleep) and 77 SNPs for continuous sleep duration. The continuous sleep duration trait has a genome‐wide genetic correlation with both short‐sleep (*r*_g_ = −0.89) and long‐sleep (*r*_g_ = 0.68) duration; the corresponding correlation between short sleep and long sleep is modest (*r*_g_ = −0.28).^16^ All instrumental variables for each trait were harmonized so that the effect alleles reflected the allele associated with an increased probability or level of exposure. Details of the SNPs used as instrumental variables and their associations with cancer are available in Supplementary Table 1.

### 2.3 Statistical analyses

The analyses were performed using the mrrobust^18^ package in Stata (version 14.2; StataCorp, College Station, Texas), and all statistical tests were two‐sided. The random‐effects inverse‐variance weighted (IVW) method was used in the main analyses. Ratio estimates were calculated for each SNP as the beta coefficient for the SNP‐cancer association divided by the beta coefficient for the SNP‐sleep duration trait association. These estimates were then combined across SNPs in a random‐effects IVW meta‐analysis. The IVW method provides the highest precision but does not correct for pleiotropic bias if present.^19^ For possible associations (*P* value <.05, IVW method), sensitivity analyses using the weighted median and MR‐Egger approaches were conducted.^19^ The weighted median method provides consistent estimates if at least 50% of the weight in the analysis comes from valid instrumental variables.^19^ The MR‐Egger approach can detect and adjust for directional pleiotropy but suffers from low power.^19^

Additionally, we conducted a sensitivity analysis omitting sleep‐related SNPs in the *FTO* gene (one SNP for the long sleep duration trait and another SNP for the continuous sleep duration trait), which has pleiotropic effects with, for example, body mass index. In a separate multivariable analysis, we additionally adjusted for smoking initiation, that is, the obtained estimates represent the direct effect of sleep duration, which is not driven by smoking initiation. Summary statistics for data for smoking initiation were obtained from the published GWAS.^20^ In the recent MR study on the link between sleep traits and risk of breast cancer in UK Biobank population, no association between the sleep trait duration allele scores and other confounding factors (eg, physical activity and alcohol consumption) was observed.^21^ As the genetic associations with sleep duration and cancer were assessed in the same population, the overestimation of genetic effect sizes (winner's course bias) may occur. In the replication stage, we performed two‐sample MR analyses of associations found in the IVW models based on UK Biobank, using an independent GWAS dataset from the FinnGen consortium.

Reported odds ratios (OR) with their 95% confidence intervals (CI) are per genetically predicted one‐unit increase in log odds of short‐ and long‐sleep duration and per genetically predicted additional hour of sleep in every 24 hours. The Benjamini‐Hochberg method was applied in the main analysis based on the UK Biobank data to correct for multiple testing of all 69 associations. *P* values that passed a critical value corresponding to false discovery rate (FDR) of .05 were considered as strong evidence of associations. *P* values that did not pass a critical value but were less than .05 were considered as suggestive evidence of associations.

## 3 RESULTS

**FIGURE 1:**
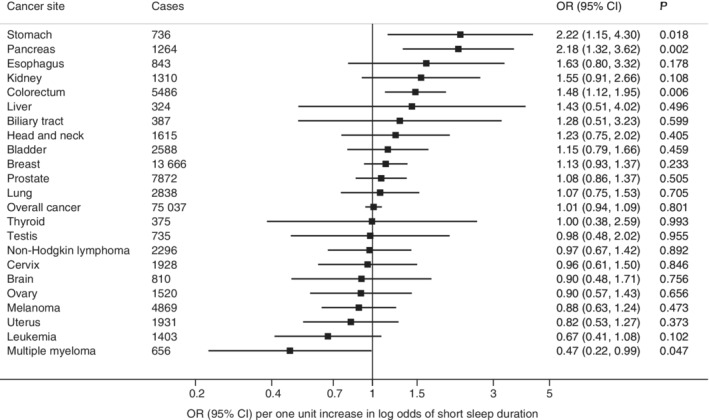
Associations of genetic liability to short‐sleep duration with overall cancer and 22 site‐specific cancers. Odds ratios are per one‐unit increase in log odds of short‐sleep duration. None of these associations passed a critical *P* value corresponding to FDR of 0.05. FDR, false discovery rate

**FIGURE 2:**
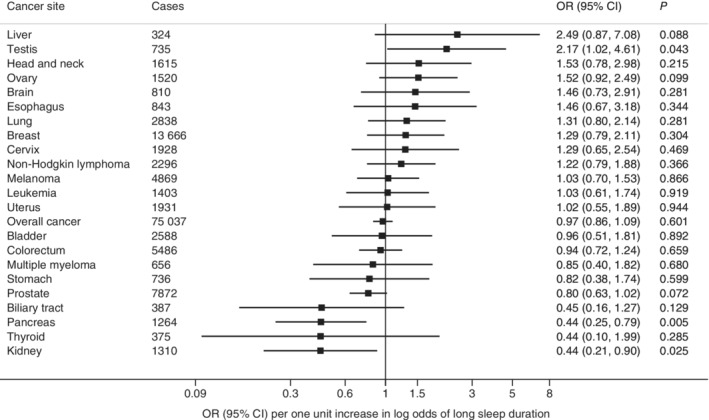
Associations of genetic liability to long‐sleep duration with overall cancer and 22 site‐specific cancers. Odds ratios are per one‐unit increase in log odds of long‐sleep duration. None of these associations passed a critical *P* value corresponding to FDR of 0.05. FDR, false discovery rate

There was no statistically significant association of genetic liability with short‐ or long‐sleep duration (Figures 1 and 2) or genetically predicted sleep duration (Supplementary Figure 1) with overall or any site‐specific cancer after correcting for multiple testing. However, there were suggestive associations of genetic liability to short sleep duration with higher odds of cancers of the stomach (OR, 2.22; 95% CI, 1.15‐4.30; *P* = .018), pancreas (OR, 2.18; 95% CI, 1.32‐3.62; *P* = .002) and colorectum (OR, 1.48; 95% CI, 1.12‐1.95; *P* = .006), but with lower odds of multiple myeloma (OR, 0.47; 95% CI, 0.22‐0.99; *P* = .047). Genetic liability to long‐sleep duration was associated with lower odds of pancreatic cancer (OR, 0.44; 95% CI, 0.25‐0.79; *P* = .005) and kidney cancer (OR, 0.44; 95% CI, 0.21‐0.90; *P* = .025) and with higher odds of testicular cancer (OR, 2.17; 95% CI, 1.02‐4.61; *P* = .043). Genetically predicted continuous sleep duration showed a suggestive inverse association with kidney cancer (OR, 0.50; 95% CI, 0.25‐0.99; *P* = .046). Removing the SNP in the *FTO* gene did not alter the observed associations except for testicular cancer (Supplementary Table 2). Likewise, the associations remained essentially the same after adjustment for smoking (Supplementary Table 2). The weighted median analysis showed similar but less precise estimates, and no directional pleiotropy was detected in the MR‐Egger analysis for most observed associations except testicular cancer (Supplementary Table 2).

Replication analyses based on the FinnGen consortium did not reveal significant associations of sleep duration traits with any cancer site from the discovery stage (Supplementary Table 3). The associations of genetically predicted short sleep with colorectal cancer (OR, 1.24; 95% CI, 0.76‐2.04; *P* = .388) and long sleep with pancreatic cancer (OR, 0.57; 95% CI, 0.17‐1.88; *P* = .354) were in the same direction as in UK Biobank.

## 4 DISCUSSION

This MR study showed suggestive evidence of a causal association between both short‐ and long‐sleep duration and risk of some site‐specific cancers but not overall cancer. After applying a multiple testing correction, genetic liability to short sleep duration was linked to nonsignificantly higher odds of several gastrointestinal cancers, including stomach, pancreatic and colorectal cancers, but with lower odds of multiple myeloma. Genetic liability to long sleep duration was associated with nonsignificantly lower odds of pancreatic and kidney cancers.

To the best of our knowledge, no previous MR study has investigated the association between genetically predicted short‐ and long‐sleep duration with overall cancer or various cancer sites. Epidemiological studies of associations of sleep duration and risk of site‐specific cancers, other than breast cancer, are scarce and inconsistent.^3^ A recent meta‐analysis of 25 observational cohort and case‐control studies indicated that neither short‐sleep nor long‐sleep duration was associated with overall cancer risk.^3^ However, long‐sleep (vs normal sleep) duration was associated with an increased risk of colorectal cancer based on six studies (OR 1.21; 95% CI 1.08‐1.34).^3^ It should be noted that there was substantial heterogeneity in this meta‐analysis.^3^ The discrepancy in results could be explained by a low number of site‐specific cancer cases in some studies, reverse causation bias, residual confounding or reporting bias. In addition, the definition of short‐ and long‐sleep duration varied among studies.

Our findings for short‐sleep duration and gastrointestinal cancers partly support the results from a recent prospective study of 297 185 adults that observed an increased risk of stomach cancer in men (hazard ratio 1.29; 95% CI 1.05‐1.59; 409 cases among 173 327 men) who reported sleep duration of 5 to 6 hours per night compared with those who slept 7 to 8 hours.^8^ In addition, our findings are in agreement with those of a case‐control study involving 1240 participants undergoing routine screening colonoscopy, which showed that short sleep duration was associated with higher odds of colorectal adenoma.^22^ In another study, comprising 75 828 postmenopausal women (851 incident cases), short (≤5 hours) and long (≥9 hours) sleep duration was associated with 36% and 47% increased risk of colorectal cancer, respectively, suggesting a U‐shaped association.^5^

In contrast to several prospective studies,^5^, ^23^ we did not find evidence for an adverse effect of long‐sleep duration on risk of cancer. If anything, a protective effect of long‐sleep duration on kidney and pancreatic cancers was observed.

There are several potential pathways that can explain the role of sleep in cancer development. Sleep duration may be a surrogate for exposure to light at night and be closely related to melatonin production, a hormone mainly produced by the pineal gland primarily during darkness. Experimental evidence suggests that besides the important role of melatonin in regulation of circadian rhythms, it is also involved in inhibition of tumor development of a wide variety of cancers, including stomach,^24^ colon^25^ and pancreatic cancers.^26^ Furthermore, melatonin exhibits anti‐inflammatory, antiangiogenic and antioxidant properties and may promote DNA repair.^4^, ^24^, ^27^ Due to its anticancer properties, melatonin has been proposed as a potential candidate for the prevention and treatment of cancer.^27^

Another potential pathway of the relationship between short sleep and gastrointestinal cancers is that excessive body weight, insulin resistance, type 2 diabetes, altered gut microbiota, inflammation and impaired immune function may mediate this association. For instance, several experimental studies have demonstrated that acute sleep deprivation may cause an alteration in glucose and insulin metabolism^28^ and hormones involved in appetite regulation, such as increased ghrelin levels (hormone produced by the gastrointestinal tract) and decreased leptin levels.^29^ Furthermore, sleep restriction was shown to increase caloric intake and unhealthy food choices^30^ as well as to alter gut microbiota.^31^ These findings are consistent with epidemiological evidence linking insufficient sleep with increased risk of obesity and type 2 diabetes,^32^, ^33^ which are associated with carcinogenesis. Moreover, sleep deprivation may indirectly, via insulin metabolism, affect circulating levels of insulin‐like growth factor‐1, which were positively associated with colorectal cancer in a previous MR study.^34^ Additionally, recent evidence suggests that sleep deprivation and sleep disorders may lead to decreased concentrations of the circulating anti‐aging protein Klotho,^35^, ^36^ which is a tumor suppressor and modulator of insulin‐like growth factor‐1 and other oncogenic signaling pathways.^37^Alterations in normal sleep patterns are further linked to gastrointestinal diseases such as gastroesophageal reflux disease, inflammatory bowel disease and ulcer,^38^ which may predispose an individual to the development of gastrointestinal cancer. With regard to multiple myeloma, its risk factor obesity is associated with sleep apnoea and chronic intermittent hypoxia, which has been recognized as a promoter of multiple myeloma.^39^ Shorter sleep duration in these settings may reduce the period of time spent under a chronic intermittent hypoxic state each night and may thus be protective for multiple myeloma risk.

A strength of this study is the MR design, which reduces confounding and reverse causality. In addition, we could assess the association between sleep duration and overall cancer in a large cohort, thereby providing high power to detect a weak association. The restriction of the population to European‐descent individuals minimized stratification bias. A limitation of this study is that the precision of the results was low or modest in analyses of site‐specific cancers and we are unable to discount the role of chance underlying these findings. Both the sleep‐related traits and cancer outcomes were assessed in a single population (UK Biobank), which might have resulted in bias toward the direction of the observational association between sleep duration and cancer. We could not replicate the results using the FinnGen data. This disparity in results could potentially be related to overestimation of the associations in UK Biobank or the smaller number of cases in FinnGen. In addition, due to a genome‐wide genetic correlation of three sleep traits used in the MR, there is an overlap in variants between these genetic instrumental variables^16^ which may increase the likelihood of chance findings. Thus, our results require confirmation by other large MR studies.

In conclusion, this MR study does not provide strong evidence to support causal associations of sleep duration with risk of overall and site‐specific cancers. The suggestive associations of short‐ or long‐sleep duration with certain cancers merit further investigation in other large MR studies.

## CONFLICT OF INTEREST

The authors declare no conflict of interest.

## ETHICS STATEMENT

Studies included in the consortia were approved by local research ethics committees and all participants provided written informed consent. The analyses were approved by the Swedish Ethical Review Authority.

## Supporting information


**Appendix** **S1:** Supplementary Material

## Data Availability

The UK Biobank data are available through the UK Biobank Access Management System (http://www.ukbiobank.ac.uk/). FinnGen data are available through online application. Summary‐level data used for the study will be made available upon reasonable request to authors.
