# Supplementary material for: Sleep duration and risk of overall and 22 site‐specific cancers: A Mendelian randomization study
Source: Int J Cancer. 2020 Sep 14;148(4):914–20. doi: 10.1002/ijc.33286 (PMC7821333; doi:10.1002/ijc.33286)
Supplement: Supplementary file 1 — Appendix S1: Supplementary Material [file IJC-148-914-s001.pdf]

SUPPLEMENTARY MATERIAL

Sleep duration and risk of overall and 22 site-specific cancers: a Mendelian randomization study

Olga E Titova[1], Karl Michaëlsson[1], Mathew Vithayathil[2], Amy M Mason[3], Siddhartha Kar,[4] Stephen Burgess[3,5], Susanna C Larsson[1,6]*

Table of contents:

Supplementary Table 1 p 2-6

Supplementary Table 2 p 7

Supplementary Table 3 p 8

Supplementary Figure 1. p 9

Supplementary table 1. Details of the single nucleotide polymorphisms used as instrumental variables and their associations with overall cancer

					Association with sleep traits				Association with overall cancer			
**Trait**	**SNP**	**Chr**	**Effect alllele**	**Other allele**	**Beta**	**SE**	**p value**		**Beta**	**SE**	**p value**	**Nearby genes**
Short sleep	rs12567114	1	G	A	0.036	0.007	4.10E-09		0.008	0.007	0.260	DPYD
Short sleep	rs2186122	1	T	A	0.024	0.006	4.80E-09		0.002	0.006	0.707	PDE4B
Short sleep	rs2820313	1	G	A	0.031	0.006	2.30E-09		0.008	0.006	0.219	LMOD1, IPO9, NAV1, SHISA4
Short sleep	rs7524118	1	C	T	0.030	0.006	4.90E-08		-0.001	0.007	0.905	CSMD2, C1orf94, GJB5
Short sleep	rs1380703	2	G	A	0.035	0.006	1.60E-11		-0.005	0.006	0.388	VRK2, LOC647016, LOC100131953
Short sleep	rs2863957	2	C	A	0.054	0.007	2.60E-18		0.002	0.007	0.812	PAX8, LOC100130100
Short sleep	rs75539574	2	A	C	0.045	0.011	8.40E-11		0.004	0.011	0.702	LINC01122
Short sleep	rs2014830	3	C	T	0.030	0.006	2.70E-08		-0.007	0.007	0.298	RBM5, and more
Short sleep	rs13107325	4	T	C	0.075	0.011	2.50E-13		0.022	0.011	0.046	SLC39A8
Short sleep	rs17005118	4	A	G	0.030	0.007	2.50E-09		0.002	0.007	0.822	RASGEF1B, PRKG2
Short sleep	rs12518468	5	C	T	0.031	0.006	8.50E-09		-0.005	0.006	0.443	ADCY2, POLS, LOC100130063
Short sleep	rs3776864	5	A	C	0.031	0.006	1.70E-08		-0.003	0.006	0.668	PAM, PPIP5K2, GIN1, c5orf30
Short sleep	rs4585442	5	G	A	0.031	0.006	8.10E-10		-0.007	0.006	0.280	SMAD5, SMAD5-AS1, TRPC7
Short sleep	rs12661667	6	T	C	0.028	0.007	2.80E-08		0.005	0.007	0.494	USP49
Short sleep	rs142180737	6	C	T	0.154	0.032	4.40E-09		-0.065	0.033	0.048	ZSCAN12, ZSCAN31, ZKSCAN3
Short sleep	rs9321171	6	C	T	0.031	0.006	4.20E-08		0.002	0.006	0.799	LAMA2, ARHGAP18
Short sleep	rs9367621	6	T	A	0.024	0.006	1.60E-08		-0.005	0.006	0.442	HCRTR2, GFRAL
Short sleep	rs11763750	7	G	A	0.035	0.008	5.10E-09		0.005	0.008	0.548	MAD1L1
Short sleep	rs1229762	7	T	C	0.037	0.003	1.10E-12		-0.003	0.006	0.595	FOXP2
Short sleep	rs60882754	8	A	T	0.055	0.012	1.80E-08		-0.008	0.012	0.511	PCMTD1, PXDNL
Short sleep	rs1607227	11	G	T	0.031	0.007	1.50E-09		0.002	0.007	0.766	METT5D1, OR2BH1P
Short sleep	rs7939345	11	T	G	0.035	0.007	4.00E-08		0.002	0.007	0.808	PTPRJ, and more

Short sleep	rs17388803	15	C	A	0.053	0.010	6.50E-10		-0.009	0.010	0.362	SEMA6D
Short sleep	rs59779556	16	T	G	0.025	0.006	2.00E-08		-0.003	0.006	0.674	GNAO1, AMFR
Short sleep	rs205024	17	C	T	0.031	0.006	2.70E-08		0.010	0.006	0.100	SHISA6
Short sleep	rs12963463	18	C	T	0.029	0.006	1.90E-11		0.005	0.007	0.459	TCF4
Short sleep	rs5757675	22	G	T	0.034	0.007	2.70E-09		-0.001	0.007	0.866	MGAT3, SYNGR1, TAB1
Long sleep	rs7534398	1	A	T	0.047	0.012	2.10E-08		-0.011	0.007	0.156	CAMTA1, VAMP3, PER3, UTS
Long sleep	rs6737318	2	G	A	0.076	0.011	3.40E-13		-0.002	0.007	0.755	PAX8, LOC100130100
Long sleep	rs549961083	5	T	C	0.534	0.117	9.60E-09		-0.021	0.083	0.801	RAB3C, PDE4D
Long sleep	rs10899257	11	A	G	0.068	0.013	4.60E-08		-0.001	0.008	0.940	GUCY2E, LRRC32, TSKU
Long sleep	rs3751046	11	G	A	0.070	0.013	2.00E-08		-0.022	0.008	0.008	BSX, C11orf63
Long sleep	rs75458655	11	T	C	0.185	0.029	5.40E-12		-0.020	0.020	0.324	MPZL2, JAML, MPZL3
Long sleep	rs17817288	16	A	G	0.039	0.009	8.90E-09		0.004	0.006	0.463	FTO, RPGRIP1L
Long sleep	rs17688916	17	T	A	0.071	0.012	1.10E-11		0.017	0.008	0.020	CRHR1, KIAA1267, LOC644246
Sleep duration	rs12567114	1	A	G	0.890	0.152	4.30E-09		-0.008	0.007	0.260	DPYD
Sleep duration	rs269054	1	A	T	0.819	0.138	2.10E-09		0.005	0.006	0.397	DAB1
Sleep duration	rs61796569	1	T	C	0.927	0.154	1.50E-09		-0.005	0.007	0.451	PDE4B
Sleep duration	rs915416	1	C	G	1.156	0.150	9.90E-15		0.000	0.007	0.988	CSMD2, C1orf94, GJB5
Sleep duration	rs10173260	2	C	T	0.770	0.139	2.90E-08		0.005	0.006	0.405	MAP2
Sleep duration	rs11885663	2	T	C	0.973	0.157	8.60E-10		-0.003	0.007	0.696	SCN1A, GALNT3, TTC21B
Sleep duration	rs12611523	2	A	G	0.758	0.137	3.10E-08		0.015	0.006	0.012	SPOPL
Sleep duration	rs374153	2	C	T	1.057	0.186	9.10E-09		-0.008	0.008	0.342	SLC8A1
Sleep duration	rs4128364	2	C	T	0.876	0.143	1.40E-09		0.010	0.006	0.104	PABPCP2, LOC100133235
Sleep duration	rs4538155	2	T	C	0.779	0.142	3.60E-08		0.007	0.006	0.266	NR4A2, GPD2
Sleep duration	rs62120041	2	T	C	1.567	0.274	9.60E-09		-0.011	0.012	0.375	MBOAT2
Sleep duration	rs72804080	2	G	A	1.068	0.192	2.90E-08		0.012	0.008	0.138	LINC01122, LOC101927285
Sleep duration	rs75539574	2	C	A	2.175	0.244	6.90E-19		-0.004	0.011	0.702	VRK2, LINC01122
Sleep duration	rs7556815	2	A	G	2.443	0.164	1.30E-49		-0.002	0.007	0.836	PAX8, LOC100130100

Sleep duration	rs112230981	3	A	G	1.892	0.314	2.20E-09		-0.021	0.014	0.121	ERC2
Sleep duration	rs13088093	3	G	T	0.976	0.144	7.00E-12		0.000	0.006	0.957	PPP2R3A, PCCB, STAG1, MSL2
Sleep duration	rs17732997	3	C	G	0.776	0.137	1.20E-08		-0.005	0.006	0.403	FOXP1, LOC100128160
Sleep duration	rs7616632	3	T	G	0.792	0.136	4.30E-09		-0.003	0.006	0.569	IL20RB, NPM1P17
Sleep duration	rs7644809	3	T	C	0.784	0.138	1.60E-08		-0.007	0.006	0.229	BBX, LOC285205
Sleep duration	rs13109404	4	T	G	1.872	0.264	1.40E-12		-0.011	0.012	0.342	BANK1
Sleep duration	rs17427571	4	A	G	0.830	0.146	1.30E-08		0.002	0.006	0.745	PRKG2
Sleep duration	rs2192528	4	A	G	0.802	0.136	2.70E-09		0.011	0.006	0.076	LCORL, LOC645174
Sleep duration	rs35531607	4	C	T	0.770	0.136	1.50E-08		0.001	0.006	0.904	CCSER1
Sleep duration	rs11567976	5	T	C	0.768	0.137	2.10E-08		0.007	0.006	0.256	CDC25C, EGR1, ETF1, GFRA3, HSPA9, NME5, CDC23, KIF20A, BRD8, FAM53C, REEP2, KDM3B
Sleep duration	rs151014368	5	A	G	0.966	0.169	9.10E-09		-0.003	0.007	0.645	LMAN2, FGFR4, SLC34A1, RGS14, PRELID1, NSD1, MXD3
Sleep duration	rs180769	5	T	C	0.763	0.138	2.30E-08		0.004	0.006	0.520	TRPC7, SMAD5, SMAD5-AS1
Sleep duration	rs365663	5	A	G	0.878	0.137	1.00E-10		0.007	0.006	0.278	SLC6A3
Sleep duration	rs460692	5	C	T	1.263	0.200	3.60E-10		-0.008	0.009	0.367	LINC01377, LOC100132531, LOC285577
Sleep duration	rs56372231	5	T	C	1.017	0.144	2.20E-12		0.003	0.006	0.604	PAM, PPIP5K2, GIN1, C5ORF30
Sleep duration	rs113113059	6	T	C	0.968	0.164	8.40E-09		-0.001	0.007	0.909	CUL9, MEA1, PPP2R5D, SMAD5-AS2, SRF, CUL7, DNPH1, MRPL2, TTBK1, RRP36, KLC4, KLHDC3
Sleep duration	rs2231265	6	G	A	0.897	0.162	2.70E-08		0.001	0.007	0.870	PNRC1
Sleep duration	rs34556183	6	A	G	1.015	0.151	2.30E-11		0.009	0.007	0.174	ZBED9, SCAND3, LOC646160,
Sleep duration	rs80193650	6	G	A	1.010	0.184	4.10E-08		0.002	0.008	0.829	ZBTB9, KIFC1, PHF1, SYNGAP1, CUTA
Sleep duration	rs9345234	6	C	A	0.781	0.138	1.80E-08		0.000	0.006	0.978	LOC100129847, LOC100128159
Sleep duration	rs9382445	6	T	C	0.872	0.140	4.80E-10		-0.004	0.006	0.470	FAM83B
Sleep duration	rs2079070	7	C	G	1.053	0.154	7.50E-12		-0.006	0.007	0.409	FOXP2
Sleep duration	rs34731055	7	T	C	1.168	0.177	3.70E-11		-0.003	0.008	0.706	MAD1L1

Sleep duration	rs7806045	7	T	C	0.887	0.158	1.40E-08		0.016	0.007	0.023	CHCHD3
Sleep duration	rs330088	8	C	T	0.868	0.137	2.70E-10		0.008	0.006	0.194	PPP1R3B, LOC100129150
Sleep duration	rs73219758	8	G	A	0.984	0.150	5.60E-11		0.013	0.007	0.052	SGCZ
Sleep duration	rs10973207	9	T	G	1.226	0.187	6.00E-11		0.002	0.008	0.850	EBLN3, ZCCHC7
Sleep duration	rs10761674	10	C	T	0.740	0.136	4.20E-08		-0.002	0.006	0.695	EGR2, ADO
Sleep duration	rs11190970	10	G	A	0.923	0.169	4.60E-08		-0.015	0.007	0.042	BTRC, FGF8, NFKB2, PITX3, PSD, FBXW4, GBF1, LDB1, NOLC1, NPM3, MGEA5, PPRC1 POLL, KCNIP2 CUEDC2, FBXL15, C10ORF76, HPS6, C10orf95, ELOVL3
Sleep duration	rs12246842	10	A	G	0.804	0.136	3.90E-09		0.001	0.006	0.914	MLLT10, DNAJC1, SKIDA1
Sleep duration	rs7915425	10	T	C	1.144	0.179	2.00E-10		0.000	0.008	0.992	BUB3, LOC100131719, GPR26
Sleep duration	rs1057703	11	G	T	1.164	0.192	1.10E-09		-0.023	0.008	0.007	BSX, C11orf63
Sleep duration	rs11602180	11	C	T	1.095	0.184	2.30E-09		0.009	0.008	0.237	PTPRJ, OR4X2, OR4B1, OR4S1, OR4X1
Sleep duration	rs1263056	11	A	G	0.768	0.137	2.00E-08		-0.012	0.006	0.051	BUD13
Sleep duration	rs12791153	11	T	A	1.413	0.253	1.90E-08		0.016	0.011	0.146	LOC729790, LOC646195
Sleep duration	rs1517572	11	C	A	0.879	0.138	1.50E-10		-0.003	0.006	0.666	METT5D1, OR2BH1P
Sleep duration	rs1553132	11	G	A	0.870	0.155	2.50E-08		-0.014	0.007	0.034	GRM5
Sleep duration	rs174560	11	C	T	0.815	0.146	2.80E-08		-0.010	0.006	0.121	FADS1, FADS3, RAB3IL1, FADS2, MYRF,V27 TMEM258, FEN1
Sleep duration	rs1939455	11	G	T	1.226	0.214	1.20E-08		0.002	0.009	0.821	TRPC6
Sleep duration	rs4592416	11	G	A	0.881	0.136	9.30E-11		0.001	0.006	0.890	HSD17B12
Sleep duration	rs7115226	11	A	C	1.594	0.261	1.70E-09		-0.009	0.011	0.410	DRD2
Sleep duration	rs7951019	11	G	T	2.213	0.391	1.20E-08		0.013	0.017	0.462	KMT2A, ARCN1, IFT46, TMEM25
Sleep duration	rs11614986	12	A	G	0.983	0.177	2.70E-08		0.003	0.008	0.701	MMAB, MVK, KCTD10, UBE3B, MYO1H
Sleep duration	rs34354917	12	C	A	0.825	0.150	3.90E-08		-0.005	0.007	0.449	ALG10B, CPNE8
Sleep duration	rs4767550	12	G	A	0.858	0.139	6.30E-10		0.003	0.006	0.642	KSR2

Sleep duration	rs10483350	14	G	A	1.042	0.172	1.50E-09		-0.007	0.008	0.333	MIR548AI, LOC100128215, PRKD1
Sleep duration	rs11621908	14	C	T	1.446	0.250	5.60E-09		0.029	0.011	0.008	ADCK1, FRDAP
Sleep duration	rs55658675	14	C	T	0.788	0.142	2.00E-08		-0.007	0.006	0.293	MAX, FNTB, GPX2, CHURC1, RAB15
Sleep duration	rs61985058	14	T	C	1.116	0.194	1.30E-08		0.002	0.008	0.815	RTN1
Sleep duration	rs6575005	14	T	C	0.934	0.159	4.40E-09		0.005	0.007	0.499	NOVA1
Sleep duration	rs8038326	15	A	G	0.955	0.152	2.80E-10		0.002	0.007	0.729	SEMA6D
Sleep duration	rs11643715	16	G	C	0.834	0.150	3.20E-08		0.003	0.007	0.683	PRKCB
Sleep duration	rs3095508	16	C	A	0.921	0.138	3.10E-11		-0.009	0.006	0.134	RBFOX1
Sleep duration	rs8050478	16	G	A	0.960	0.136	1.70E-12		0.002	0.006	0.729	GNAO1
Sleep duration	rs9940646	16	C	G	1.017	0.137	1.20E-13		0.010	0.006	0.084	FTO
Sleep duration	rs1991556	17	G	A	0.994	0.163	1.00E-09		0.014	0.007	0.043	MAPT, STH, KANSL1, LOC644157, LOC644172
Sleep duration	rs205024	17	T	C	0.830	0.140	3.90E-09		-0.010	0.006	0.100	SHISA6
Sleep duration	rs2139261	17	G	C	1.122	0.174	8.50E-11		0.000	0.008	0.977	KCNJ12
Sleep duration	rs7503199	17	C	T	0.885	0.154	1.00E-08		0.010	0.007	0.155	PER1, PFAS, SMAD5-AS5, VAMP2, AURKB, ARHGEF15, RANGRF, BORCS6, CTC1, TMEM107, KRBA2, SLC25A35
Sleep duration	rs9903973	17	C	T	0.766	0.136	2.60E-08		0.002	0.006	0.760	CA10, LOC339209
Sleep duration	rs12607679	18	T	C	1.208	0.156	8.30E-15		-0.001	0.007	0.935	TCF4
Sleep duration	rs10421649	19	A	T	0.798	0.138	6.90E-09		0.004	0.006	0.477	FBXL12, UBL5, PIN1
Sleep duration	rs2072727	20	T	C	0.795	0.137	7.90E-09		-0.002	0.006	0.749	YWHAB, PABPC1L

Supplementary table 2. Association between genetically predicted sleep duration and cancer in the primary inverse-variance weighted analysis and in sensitivity analyses using other MR methods and excluding SNPs in the FTO gene region

		**IVW, univariable**		**IVW, adjusting for smoking***		**IVW, removing SNPs in *FTO* gene****		**Weighted median**		**MR-Egger estimate**		**MR-Egger intercept**	
**Cancer site**	**Trait**	**OR (95% CI)**	**p value**	**OR (95% CI)**	**p value**	**OR (95% CI)**	**p value**	**OR (95% CI)**	**p value**	**OR (95% CI)**	**p value**	**Intercept**	**p value**
Kidney	Long sleep	0.44 (0.21-0.90)	0.025	0.45 (0.21-0.94)	0.033	0.43 (0.19-0.97)	0.043	0.33 (0.15-0.76)	0.009	0.18 (0.03-1.26)	0.085	0.063 (-0.066 to 0.191)	0.340
Kidney	Sleep duration	0.50 (0.25-0.99)	0.046	0.53 (0.26-1.07)	0.076	0.48 (0.24-0.97)	0.041	0.53 (0.21-1.35)	0.184	0.17 (0.01-2.38)	0.188	0.018 (-0.025 to 0.061)	0.409
Stomach	Short sleep	2.22 (1.15-4.30)	0.018	2.23 (1.15-4.34)	0.018	2.22 (1.15-4.30)	0.018	2.23 (0.91-5.44)	0.079	1.62 (0.17-15.13)	0.672	0.012 (-0.066 to 0.089)	0.772
Colorectum	Short sleep	1.48 (1.12-1.95)	0.006	1.52 (1.16-1.99)	0.002	1.48 (1.12-1.95)	0.006	1.52 (1.08-2.14)	0.017	1.74 (0.66-4.55)	0.261	-0.006 (-0.039 to 0.028)	0.73
Pancreas	Short sleep	2.18 (1.32-3.62)	0.002	2.09 (1.25-3.48)	0.005	2.18 (1.32-3.62)	0.002	1.85 (0.92-3.72)	0.085	1.46 (0.26-8.13)	0.665	0.015 (-0.045 to 0.074)	0.631
Pancreas	Long sleep	0.44 (0.25-0.79)	0.005	0.43 (0.24-0.78)	0.006	0.43 (0.23-0.78)	0.006	0.44 (0.21-0.93)	0.033	0.39 (0.09-1.65)	0.200	0.009 (-0.089 to 0.107)	0.852
Testis	Long sleep	2.17 (1.02-4.61)	0.043	2.32 (1.08-4.99)	0.031	1.89 (0.86-4.15)	0.112	2.13 (0.81-5.62)	0.125	0.27 (0.04-1.94)	0.191	0.152 (0.019-0.286)	0.025
Multiple myeloma	Short sleep	0.47 (0.22-0.99)	0.047	0.49 (0.23-1.05)	0.065	0.47 (0.22-0.99)	0.047	0.59 (0.21-1.66)	0.32	1.01 (0.07-15.02)	0.995	-0.027 (-0.120 to 0.066)	0.566

IVW, inverse-variance weighted (random-effects model); MR, Mendelian randomization; SNP, single-nucleotide polymorphism.
*Note that only 7 of 8 SNPs associated with long sleep were available in the smoking data set.
**One SNP associated with long sleep and one SNP associated with sleep duration were located in the FTO gene region; those two SNPs were excluded in this sensitivity analysis.

Supplementary table 3. Replication analyses using FinnGen of the suggestive associations in UK Biobank (p<0.05)

			IVW, univariable		Weighted median		MR-Egger estimate		MR-Egger intercept	
**Cancer site**	**Cases***	**Trait****	**OR (95% CI)**	**p value**	**OR (95% CI)**	**p value**	**OR (95% CI)**	**p value**	**Intercept**	**p value**
Stomach	329	Short sleep	0.59 (0.19-1.90)	0.382	0.64 (0.13-3.16)	0.584	0.53 (0.00-80.23)	0.803	0.004 (-0.162 to 0.170)	0.962
Colorectum	1573	Short sleep	1.24 (0.76-2.04)	0.388	1.11 (0.56-2.22)	0.762	3.50 (0.43-28.39)	0.241	-0.035 (-0.105 to 0.034)	0.319
Pancreas	367	Short sleep	0.65 (0.23-1.82)	0.414	0.51 (0.12-2.11)	0.353	4.62 (0.06-364.22)	0.492	-0.067 (-0.211 to 0.078)	0.367
Pancreas	367	Long sleep	0.57 (0.17-1.88)	0.354	0.37 (0.08-1.74)	0.207	0.02 (0.00-0.61)	0.024	0.224 (0.010 to 0.437)	0.040
Multiple myeloma	325	Short sleep	0.87 (0.29-2.62)	0.808	1.65 (0.37-7.31)	0.511	2.84 (0.02-336.4)	0.669	-0.040 (-0.198-0.118)	0.619
Kidney	526	Long sleep	1.30 (0.48-3.55)	0.604	1.05 (0.30-3.68)	0.936	3.61 (0.22-58.56)	0.367	-0.071 (-0.251-0.110)	0.443
Kidney	526	Sleep duration	1.08 (0.37-3.17)	0.888	0.86 (0.16-4.52)	0.861	1.44 (0.02-108.42)	0.861	-0.005 (-0.075 to 0.065)	0.892

IVW, inverse-variance weighted (random-effects model); MR, Mendelian randomization; SNP, single-nucleotide polymorphism.
*The number of controls (individuals without cancer) was 120,006.
**The analyses included 26 SNPs for short sleep, 7 SNPs for long sleep, and 73 SNPs for sleep duration.

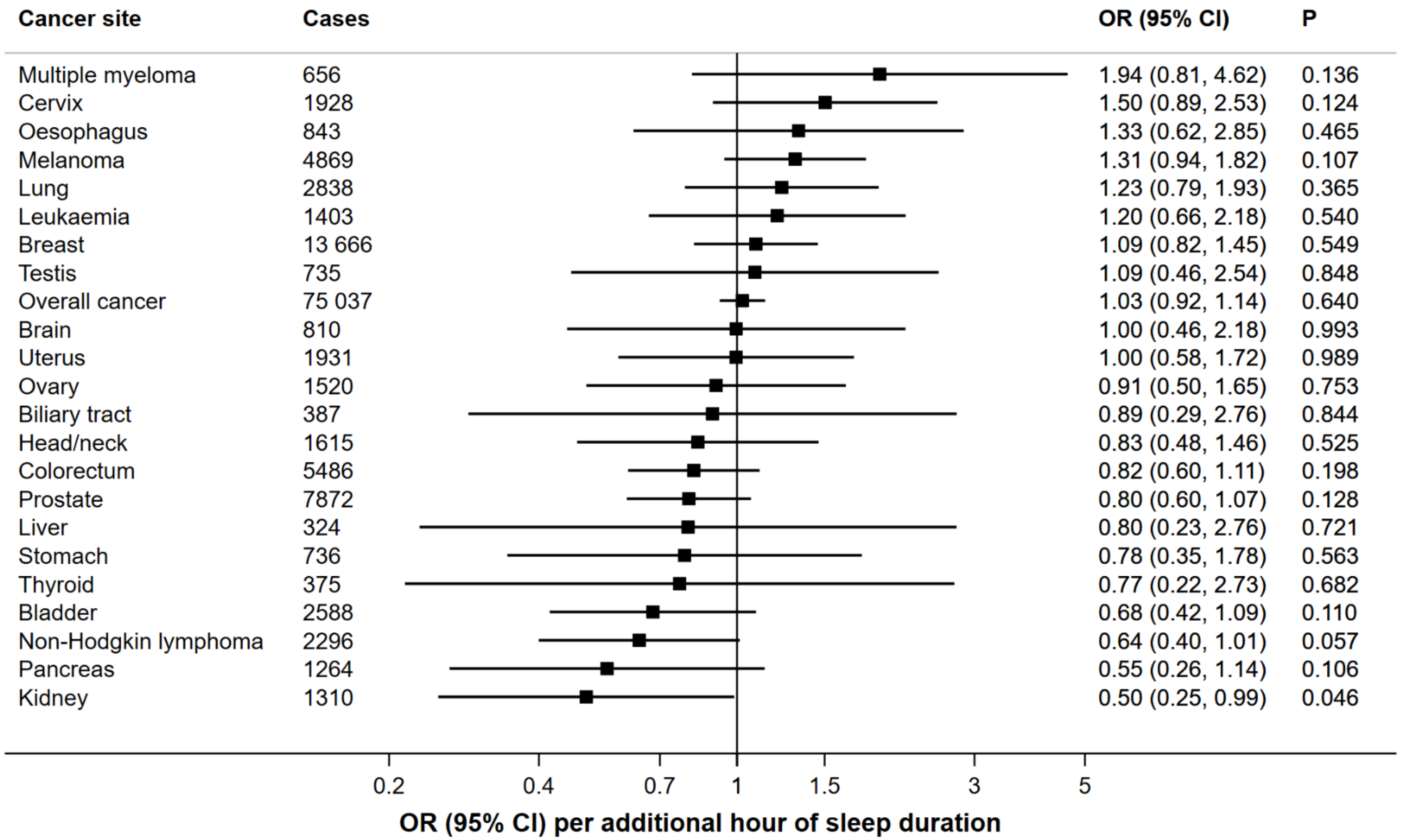

Supplementary Figure 1. Associations of genetically predicted continuous sleep duration with overall cancer and 22 site-specific cancers.

Odds ratios are per additional hour of sleep in every 24 hours.
